# Diagnostic Performance of Preoperative Choline-PET/CT in Patients Undergoing Salvage Lymph Node Dissection for Recurrent Prostate Cancer: A Multicenter Experience

**DOI:** 10.3390/tomography8020089

**Published:** 2022-04-11

**Authors:** Łukasz Nyk, Hubert Kamecki, Wojciech Krajewski, Bartosz Małkiewicz, Tomasz Szydełko, Markiian Kubis, Marcin Słojewski, Piotr Kryst, Sławomir Poletajew, Wojciech Malewski

**Affiliations:** 1 Second Department of Urology, Centre of Postgraduate Medical Education, 01-809 Warsaw, Poland; lukasz.nyk@cmkp.edu.pl (Ł.N.); piotr.kryst@bielanski.med.pl (P.K.); slawomir.poletajew@cmkp.edu.pl (S.P.); wojciech.malewski@ecz-otwock.pl (W.M.); 2 Department of Minimally Invasive and Robotic Urology, University Center of Excellence in Urology, Wrocław Medical University, 50-556 Wrocław, Poland; wojciech.krajewski@umw.edu.pl (W.K.); bartosz.malkiewicz@umw.edu.pl (B.M.); tomasz.szydelko@umw.edu.pl (T.S.); 3 Department of Urology and Urological Oncology, Pomeranian Medical University, 70-111 Szczecin, Poland; mv.kubis@gmail.com (M.K.); mslojewski@gmail.com (M.S.)

**Keywords:** positron emission tomography computed tomography, prostatic neoplasms, salvage therapy, predictive value of tests, sensitivity and specificity

## Abstract

We aimed to retrospectively analyze consecutive prostate cancer patients diagnosed with biochemical or clinical recurrence after local treatment with curative intent, with no evidence of distant metastases, who underwent positron emission tomography/computed tomography (PET/CT) with choline followed by salvage lymph node dissection (SLND) in three academic centers between 2013 and 2020. A total of 27 men were included in the analyses. Sensitivity, specificity, positive and negative predictive values, and accuracy of choline-PET/CT in predicting pathology-proven lymph node involvement were 75%, 43%, 79%, 38% and 67% on per-patient and 70%, 86%, 80%, 78%, and 79% on per-site analyses, respectively, with the differences in specificity and NPV between per-patient and per-site analyses being statistically significant (*p* = 0.03 and 0.04, respectively). The study provides further insight into the role of preoperative choline-PET/CT in patients undergoing SLND for recurrent PC.

## 1. Introduction

Biochemical recurrence (BCR) in prostate cancer (PC) patients managed with curative intent is being commonly encountered in clinical practice, as expected to occur in more than half of men with high-risk disease treated with radical prostatectomy (RP) [1]. In 82–91% of patients with BCR after RP, the location of recurrence is confined to the fossa or pelvic lymph nodes [2], making salvage lymph node dissection (SLND) a reasonable, yet still investigated treatment option in selected patients [3,4,5]. In patients fit for curative salvage treatment, a prostate specific membrane antigen (PSMA) positron emission tomography/computed tomography (PET/CT) or choline PET/CT or fluciclovine PET/CT scan is recommended by the contemporary guidelines [6], in order to assess location of recurrence. While the role of PET/CT as a superior imaging modality in PC patients experiencing BCR is considered to be promising, high-quality data to verify its diagnostic values is still lacking [7]. The aim of this study is to provide further insight into the performance of preoperative choline-PET/CT in patients undergoing SLND for recurrent PC.

## 2. Materials and Methods

After obtaining institutional ethics board approval (ID number: 120/PB/2018) we retrospectively analyzed consecutive patients who underwent salvage pelvic lymph node dissection for recurrent prostate cancer in three academic centers between 2013 and 2020. Inclusion criteria were: (i) having undergone local treatment with curative intent (radical prostatectomy or radiotherapy); (ii) having been preoperatively diagnosed with either biochemical recurrence (BCR) or clinical recurrence; (iii) having undergone preoperative imaging with positron emission tomography/computed tomography (PET/CT) radiolabeled with choline; and (iv) no distant bone or visceral metastases identified preoperatively. We defined BCR as a prostate specific antigen with a (PSA) level > 0.4 ng/mL and rising after radical prostatectomy (RP), or a PSA level greater than 2.0 ng/mL over the nadir and rising after radical radiotherapy (RT). We defined clinical recurrence as nodal involvement demonstrated on choline-PET/CT, as determined by the nuclear radiologist who read the images. Patients with missing or incomplete data were excluded from the study.

Patient data was collected from institutional medical records. Based on preoperative choline-PET/CT results and postoperative pathology reports we calculated diagnostic values: sensitivity; specificity; positive predictive value (PPV); negative predictive value (NPV); and accuracy of choline-PET/CT in predicting lymph node involvement. We performed both a per-patient and a per-site analysis. In the per-site analysis we differentiated between the right or left side, regardless of exact anatomic localization. Diagnostic values were expressed as percentages with 95-percent confidence intervals (95%-CIs), calculated as ±1.96 × standard error. For comparison of ratios, we used Fisher’s exact test. Continuous variables were compared with a Mann–Whitney test. For comparison of sensitivity, specificity, PPV, NPV and accuracy we compared ratios of true positives (TP) versus false negatives (FN), true negatives (TN) versus false positives (TP), TP versus FP, TN versus FN and TP + TN versus FP + FN, respectively. Observed outcomes were considered statistically significant when *p*-value < 0.05.

Statistical analyses were performed using PSPP software (GNU, version 1.4.1-g79ad47, Free Software Foundation, Boston, MA, USA).

## 3. Results

We identified 28 patients who met the inclusion criteria. One patient was excluded due to incomplete data in regard to the final pathology results. The remaining 27 men were included in the analyses. Baseline patient characteristics are presented in Table 1. All patients underwent imaging with 11C- or 18F-radiolabelled choline PET/CT. All SLND procedures were performed with traditional (non-robotic) laparoscopy and with the intent to remove all the pelvic lymphatic tissue.

Among 15 patients who had received RP alone as primary local treatment, five (33%) had been treated with androgen deprivation therapy (ADT) at any point before SLND. Patients who had received radiation therapy, either as primary local treatment or as adjuvant or salvage treatment after prostatectomy, were managed with adjuvant ADT in line with contemporary guidelines.

As demonstrated in Table 1, there were 12 patients who had received any form of radiotherapy before SLND. Compared to the other 15 patients, the median number of dissected lymph nodes in this group was not significantly different (10 versus 10, *p* = 0.98).

The results and comparison of per-patient and per-site analyses of the diagnostic performance of choline-PET/CT in predicting pelvic lymph node involvement are presented in Table 2.

Finally, we analyzed whether the levels of preoperative serum PSA, as well as the numbers of dissected lymph nodes differed between patients who were accurately (true positives and true negative) or non-accurately (false positives and false negatives) assessed with preoperative choline-PET/CT. The results of these comparisons can be seen in Table 3.

## 4. Discussion

In our study, we presented diagnostic values of preoperative choline-PET/CT in patients undergoing SLND for recurrent PC, with final pathology results serving as a reference.

Despite the superiority of PSMA-PET/CT over choline-PET/CT having been acknowledged in the literature [8], we decided to present data for choline-PET/CT, in order to represent a clinical scenario still typically seen in many centers.

Our findings are similar to the results presented in previously published papers. A meta-analysis by Evangelista et al. [9], in which results from eighteen studies on recurrent prostate cancer patients who underwent choline-PET/CT prior to SLND were included, reported the pooled sensitivity, specificity, PPV and NPV of choline-PET/CT to be: 85% (95% CI: 79–90%), 33% (25–41%), 75% (68–81%), and 50% (40–59%), respectively, on the patient-based analysis. These values are comparable to the results presented in our study. However, several of the studies included in the discussed meta-analysis were based on the pathology results from limited, PET/CT-directed lymph node dissection, making the extrapolation of the calculated pooled diagnostic performance to our patient cohort questionable.

Another meta-analysis by Sathianathen et al. [7], which included sixteen articles, in which 11C-choline-PET/CT was evaluated for diagnostic performance in visualizing the biochemical recurrence of prostate cancer, the summary sensitivity was calculated to be 81% (95% CI: 70–88%) on a per-patient analysis, which is comparable to our findings. While the summary specificity reported in the meta-analysis was as high as 84% (95% CI: 70–92%), which may seem to outperform the values reported in our scenario, it is important to recognize that the studies included in the meta-analysis validated the results using a combination of histology, further imaging or clinical follow-up, and many cases of negative PET/CT scans may have not been verified pathologically, which poses a significant bias to calculations of specificity.

In line with the above considerations, a risk of selection bias should not be ignored, as all the patients considered for our study were men in whom SLND was performed. There might have been a substantial number of patients, probably with negative PET/CT, who did experience BCR, but did not undergo SLND, as this is not considered a standard procedure in such a setting. Perhaps, if SLND had been performed in those patients, the specificity of choline-PET/CT would have turned out to be even lower. Moreover, there might have been a confounding factor that both led to proceeding with SLND in PET/CT-negative patients, who comprised 30% of our cohort, and influenced the pathology-verified diagnostic performance of the test.

A clinically significant conclusion can be drawn from our study, as we demonstrated significantly higher specificity and NPV of choline-PET/CT on the per-site analysis than on the per-patient analysis. The observed relationship remains in concordance with previously published studies [9,10]. The discrepancy between false negative rates on per-patient and per-site analyses might be explained by a hypothesis that while there are patients in whom the cancer is difficult to be visualized with PET/CT for various reasons, in other patients, in whom a lesion is detected, which serves as proof of the individual cancer metabolism being detectable with PET/CT, the negative results for other sites are unlikely to be false. Individual prostate cancer genetic and metabolic profiling is a relevant topic of recent investigations and soon may demonstrate an important role in reaching a personalized approach in recurrent prostate cancer patients [11]. While research on individual variations in PSMA expression leading to altered PSMA-PET/CT accuracy has been recently published in the literature [12], similar studies in regard to choline-PET/CT have not been carried up to date. Further investigation into the site-related NPV of PET/CT in patients presenting with positive scans could be of aid in the possible development of lesion-targeted salvage therapies in selected patients.

Despite the literature data showing that choline-PET/CT performance in recurrent prostate cancer may be influenced by the PSA level [9,13], as is the case with PSMA-PET [14], we did not evaluate for such a relationship in our study, further than demonstrating a non-significant difference between accurately and non-accurately assessed patients due to the small sample size. It is unclear whether this would impact the observed differences between the per-patient and per-site analyses.

As explained in Methods, for the purpose of the per-site analysis, we defined “site” as side (right or left), from where a node was removed, instead of as a specific lesion. The reason for doing so was the inability to retrospectively verify whether the anatomic location of the dissection site, as stated in the name of the specimen, truly and accurately represented the location reported by the nuclear radiologist who read the PET/CT images. Nevertheless, with this approach, we still demonstrated significant differences as compared to the per-patient analysis.

The median number of dissected LNs in our patients (10, interquartile range: 6–25) may be considered relatively low for a good salvage lymphadenectomy. Data from a recent systematic review indicate that other studies on patients undergoing SLND reported median numbers of dissected LNs between 6.5 and 83 [5]. While we reported that having undergone prior radiotherapy was not significantly associated with a reduced number of removed LNs in our patients, contrary to hypothetical expectations that intraoperative difficulties due to post-radiation scarring could have influenced the quality of SLND, having undergone prior extended lymphadenectomy with radical prostatectomy might have been a factor that had influenced the extent of salvage dissection (due to scarring and smaller amounts of remnant lymphatic tissue). Unfortunately, we lack data in regard to which patients underwent extended lymphadenectomy as primary treatment and how many nodes, if any, were resected at that point, which should be considered a limitation to this study. Furthermore, SLNDs in our study group were performed by a number of urologists in three centers and we lack specific data in regard to the surgeon’s expertise in each case, which could possibly serve as an additional factor for the quality of dissection, given the challenging nature of salvage lymphadenectomy after primary local treatment.

We demonstrated that the higher number of dissected LNs during SLND was significantly associated with accurate preoperative clinical assessment with choline-PET/CT. Obviously, one would expect that missing metastatic lymph nodes during SLND would decrease true positive rates (in PET/CT-positive patients), as well as increase true negative rates (in cases of negative PET/CT), either improving or impairing accuracy, respectively. While the majority of our patients (70%) were PET/CT-positive, it is reasonable to assume that the intraoperative missing of lymph nodes might have been responsible for decreased choline-PET/CT performance in our cohort. Unfortunately, while we lack a higher-tier reference than the pathology results, we are not able to verify whether this hypothesis is true. Nevertheless, our data may support the rationale of using auxiliary techniques, which have been proven to be effective in improving the efficiency of lymph node dissection in PC patients, such as indocyanine green guidance [15,16].

The main limitations of our study are its retrospective design and small sample size. Moreover, the unambiguous interpretation of data is impeded by the heterogeneity of our patient cohort. The different type and extent of primary local treatment could have had a significant impact on recurrence patterns. Furthermore, the marked differences in the number of dissected lymph nodes, as well as the other limitations discussed above, might have posed a risk of bias to the study. The advantage of the study is the use of SLND pathology reports as a reference standard.

## 5. Conclusions

While being limited mainly by retrospective design and a small sample size, our study provides further insight into the diagnostic performance of choline-PET/CT prior to SLND in recurrent prostate cancer and spotlights topics that could become subjects of research in the next future. Deeper investigation into the per-site versus per-lesion NPV of choline-PET/CT in prostate cancer patients experiencing BCR after treatment with curative intent may help in developing refined salvage treatment strategies.

## Figures and Tables

**Table 1:** Baseline patient characteristics.

Characteristic		All Patients (*n* = 27)
Median age ^a^, year (IQR)		67 (62–69)
Local treatment	RP alone	15 (56%)
	RP + ART/SRT	11 (41%)
	EBRT	1 (4%)
Grade group ^b^	1	5 (19%)
	2	7 (26%)
	3	5 (19%)
	4	7 (26%)
	5	3 (11%)
Median PSA at SLND, ng/mL (IQR)		2.34 (1.26–7.97)
Median time from LT to SLND, months (IQR)		60 (31–87)
cN+ on PET/CT		19 (70%)
Median number of dissected nodes (IQR)		10 (6, 25)
pN+ on pathology		20 (74%)
Median positive nodes density ^c^ (IQR)		32% (13–62%)

IQR—interquartile range, RP—radical prostatectomy, ART—adjuvant radiation therapy, SRT—salvage radiation therapy, EBRT—external beam radiation therapy, PSA—prostate specific antigen, SLND—salvage lymph node dissection, PET/CT—positron emission tomography/computed tomography. ^a^ Age at SLND. ^b^ Pathology grade group for RP patients, biopsy grade group for EBRT patients. ^c^ Calculated for pN+ patients only.

**Table 2:** The results and comparison of per-patient and per-site analyses of the diagnostic performance of choline-PET/CT in predicting pelvic lymph node involvement.

	Per-Patient Analysis (95% CI)	Per-Site Analysis (95% CI)	*p*-Value
Sensitivity	75% (56–94%)	70% (51–88%)	N.S.
Specificity	43% (6–80%)	86% (74–99%)	0.03
PPV	79% (61–97%)	80% (62–98%)	N.S.
NPV	38% (4–71%)	78% (64–92%)	0.04
Accuracy	67% (49–84%)	79% (68–90%)	N.S.

PET/CT—positron emission tomography/computed tomography, PSA—prostate specific antigen, PPV—positive predictive value, NPV—negative predictive value, 95% CI—95% confidence interval, N.S.—non-significant.

**Table 3:** The differences between preoperative PSA levels and the numbers of dissected lymph nodes between accurately and non-accurately assessed patients.

	Accurate Assesment (*n* = 18)	Non-Accurate Assesment (*n* = 9)	*p*-Value
Median preoperative PSA (IQR), ng/mL	3.2 (1.9–8.2)	1.3 (1.1–2.6)	N.S.
Median number of LNs dissected (IQR)	15 (9–37)	6 (5–7)	0.01

PSA—prostate specific antigen, LNs—lymph nodes, IQR—interquartile range, N.S.—non-significant.

## Data Availability

The data analyzed in this study are available upon request from the corresponding author.
